# First person – Mikaela Scheer

**DOI:** 10.1242/bio.061968

**Published:** 2025-03-25

## Abstract

First Person is a series of interviews with the first authors of a selection of papers published in Biology Open, helping researchers promote themselves alongside their papers. Mikaela Scheer is first author on ‘ Alveolar epithelial paxillin in postnatal lung alveolar development’, published in BiO. Mikaela is a research technician in the lab of Akiko Mammoto at the University of Wisconsin, USA, investigating the role of angiogenesis in lung development and regeneration.


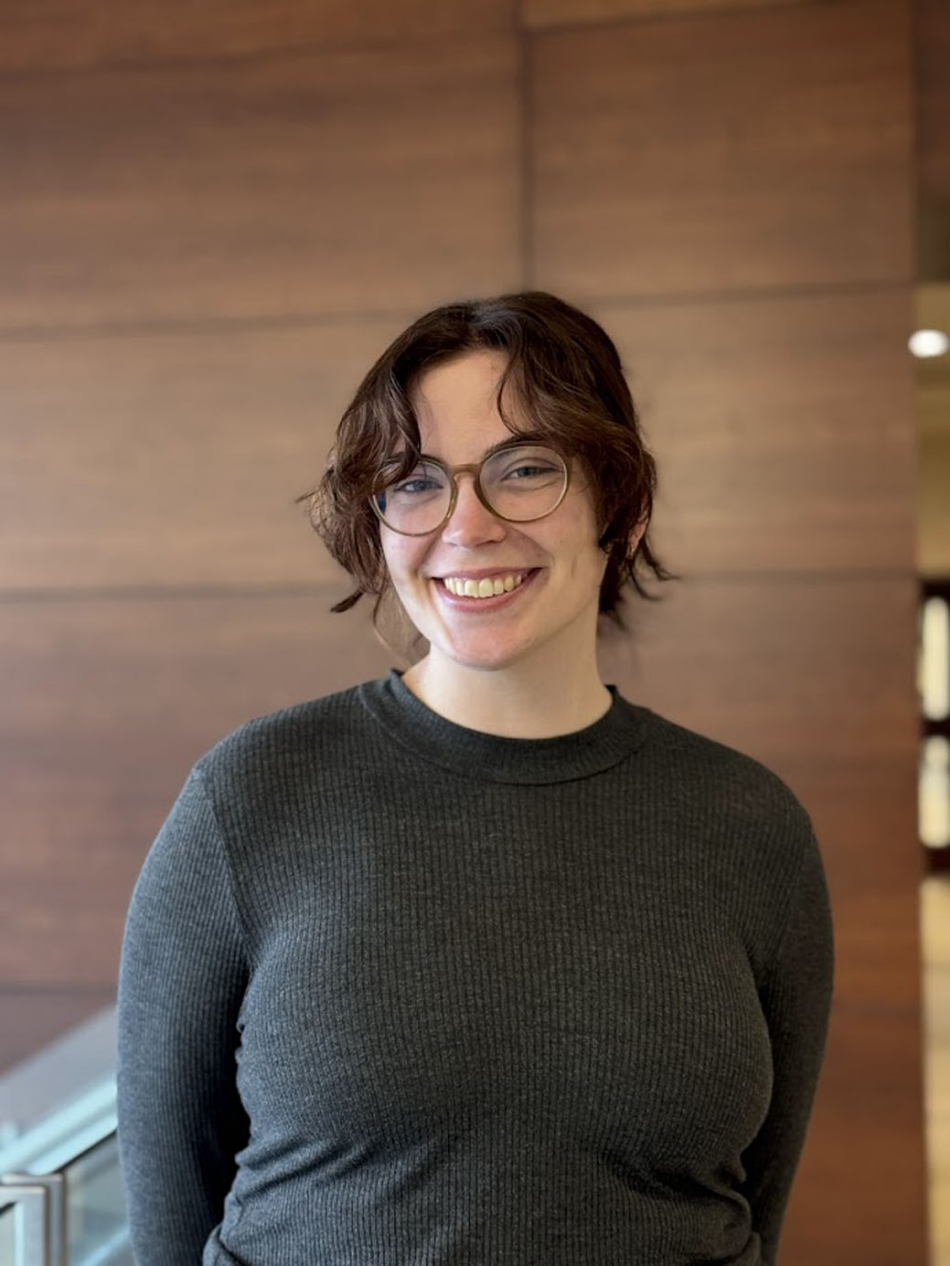


**Mikaela Scheer**

**Describe your scientific journey and your current research focus**

I have always been interested in biology, mainly evolutionary and ecological sciences. In 2023, I received my Bachelors of Science in plant/fungal biology at the University of Wisconsin – La Crosse. After I graduated, I became a research technician at the Medical College of Wisconsin where I delved into molecular biology, specifically lung development and regeneration.

My curiosity of the world around me lead me to pursue a career in science.

**Who or what inspired you to become a scientist?**

As a child, I was fascinated with nature documentaries. I loved learning about different animal species and how they interacted with each other. My curiosity of the world around me lead me to pursue a career in science.

**How would you explain the main finding of your paper?**

This research focuses on an important protein called paxillin, which plays a key role in cell growth and movement. We found that paxillin controls the development of cells in lung air sacs in mouse pups by regulating the expression of factors that stimulate expansion of air sacs and facilitate breathing.

We found that paxillin controls the development of cells in lung air sacs

**What are the potential implications of this finding for your field of research?**

Impairment of lung development is one of the most significant features of neonatal lung diseases. These findings suggest that manipulation of paxillin in the lung cells could be used as an intervention for neonatal lung diseases including bronchopulmonary dysplasia.

**Figure:**
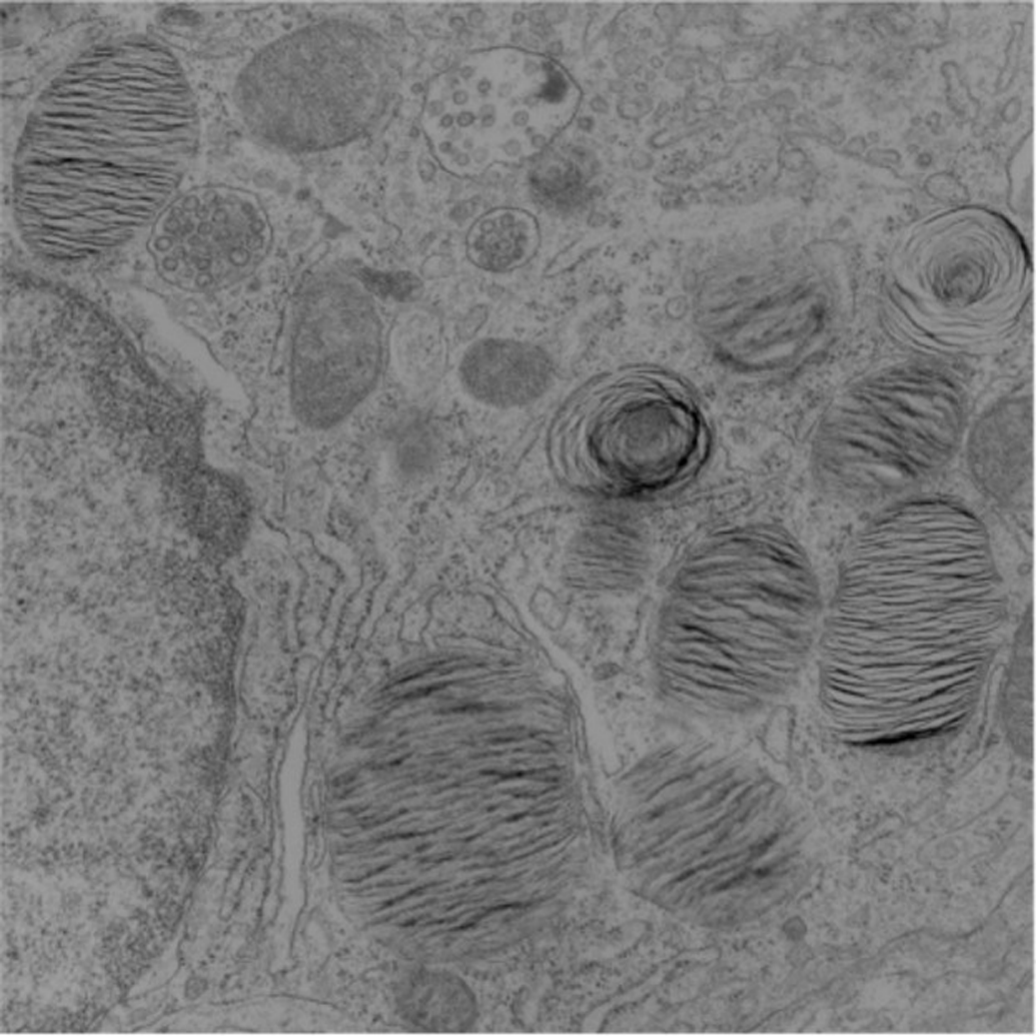
Transmission electron microscopy image of postnatal day 10 (P10) mouse lung showing the lamellar structures in lamellar bodies.

**Which part of this research project was the most rewarding?**

This was one of the first big research projects I have ever worked on. Being part of the whole process and seeing things coming to fruition was very exciting. It was especially rewarding to present these findings at internal as well as external conferences and have people being just as excited as you are about the results.

**What do you enjoy most about being an early-career researcher?**

I love how there are endless possibilities in science. Since I am just starting my career, there are so many other things to discover. It is very exciting to think of all the places science could take me.

**What piece of advice would you give to the next generation of researchers?**

A lot of big decisions are pushed onto young researchers before we really know what we enjoy doing. Most of us are expected to continue with higher education as soon as we are finished with undergrad. Because of this, I would say don't rush any big decisions unless you are 100% confident that is what you want to do. There is no harm in taking your time when it comes to deciding your next steps.

**What's next for you?**

Currently, I am a research technician, but I am looking to enter a PhD program soon. Eventually, I would like to go back to my roots and focus more on ecological research after gaining more experience in wet lab techniques.
